# Supplementary material for: Invasive Fungal Infections Complicating COVID-19: A Narrative Review
Source: J Fungi (Basel). 2021 Oct 29;7(11):921. doi: 10.3390/jof7110921 (PMC8620819; doi:10.3390/jof7110921)
Supplement: Supplementary file 1 [file jof-07-00921-s001.zip › tables/Table S1_CAPA reports.pdf]

Table S1. Case report and case series describing CAPA.

Author/ Country/ Algorithm	No. cases/ Definition	Sex/Age	Underlying diseases	Steroid / Target therapy COVID-19	Days from ICU/TI to CAPA (IQR)	Sample/ *Aspergillus* spp.	GM (ODI) index Serum/ respiratory specimen	*Aspergillus* PCR in serum/ respiratory specimen	Positive BDG	Specific imaging findings	Antifungals	Outcome
Trovato [65]/ Italy/ NR	1/NR	M/77	AH, DM	Steroid	16	BAS/*A. niger* Positive DE	4.9/-	-/Pos (BAS)	Yes	No	Voriconazole	Dead
Benedetti [66]/ Argentina/ mAspICU	5/ Probable	M/23	ALL	Steroid	6	TA/Neg	0.9/4.2 (TA)	-	-	Nodules	Voriconazole	Dead
	Probable	M/57	None	Steroid	3	Sputum/ *A. fumigatus*	0.92/-	-	-	No	AMB	Alive
	Probable	M/44	DM, AH, Obesity	Steroid	32	TA/Neg	1.01/-	-	-	Nodules	Voriconazole	Alive
	Probable	M/69	DM	Steroid	6	TA/*A. fumigatus* Positive DE	2.15/Neg (TA)	-	-	-	Voriconazole	Alive
	Probable	F/69	DM, AH	Steroid	13	TA/*A. fumigatus*	0.57/3.17 (TA)	-	-	-	Voriconazole	Alive
Iwanaga [67]/ Japan/ NR	1/NR	M/79	Polymyalgia rheumatica (chronic steroids)	Steroid	23*	TA/*A. fumigatus* Positive DE	-	-	-	Nodules	AMB	Dead
Lahmer [68]/ Germany/ NR	2/NR	M/80	CPD	-	5	BAL/ *A. fumigatus*	1.5/6.3 (BAL)	-	-	No	AMB	Dead
	NR	M/70	No	-	6	BAL/ *A. fumigatus*	Neg/6.1 (BAL)	-	-	No	AMB	Dead
Antinori [69]/ Italy/ NR	1/ Proven (autopsy)	M/73	Obesity, AH, DM, CVD	No	4	BAS/ *A. fumigatus*	8.6/-	-	-	No	Isavuconazole AMB	Dead
Prattes [70]/ Austria/ NR	1/NR	M/70	AH, DM, CVD, CPD (chronic steroids)	-	2	TA/*A. fumigatus*	Neg/-	-	Neg	Halo sign	Voriconazole	Dead
Blaize [71]/ France/ AspICU	1/Putative	M/74	AH, MDS	-	4	TA/*A. fumigatus* Positive DE	Neg/Neg (TA)	Neg/Pos (TA)	Neg	No	No	Dead
Santana [72]/ Brazil/ NR	1/Proven (autopsy)	M/71	AH, DM	-	-	Autopsy/ *A. penicillioides*	-	-	-	Nodule	No	Dead
Helleberg [73]/ Denmark/ AspICU	2/ Putative	F/63	AH	No	5	TA/*A. fumigatus*	1.1/8.2 (BAL)	-	-	No	Voriconazole	Dead
	Putative	F/53	CPD	Steroids	1	TA/*A. fumigatus*	Neg/2.2 (TA)	-	-	No	Voriconazole	Dead
Bruno [74]/ Italy/ mAspICU	1/Probable	M/68	No	TCZ	24	BAL/ *A. fumigatus*	Neg/Pos (BAL)	-	-	Cavity + air crescent	Voriconazole	Alive

Mohamed[75]/ Ireland/ mAspICU	1/Probable	M/66	Obesity, AH, DM	-	7	TA/*A. fumigatus*	1.1/5.5 (TA)	-	Pos	No	AMB	Dead
Ghelfenstein [76]/France/ AspICU	1/Putative	M/56	Obesity, DM, CPD	Eculizumab	7	TA/ *A. fumigatus* Positive DE	Neg/-	-/Pos (TA)	Neg	Nodule	No	Dead
Fernandez [77]/Argentina/ mAspICU	1/Probable	M/85	AH	Steroid	25	TA/*A. flavus*	1.4/-	-	-	No	Voriconazole AMB	Dead
Schein [78]/ France/ EORTC pers.°	1/Probable	F/87	No	Steroid	-	TA/Neg	1.9/6.03 (sputum)	Pos/-	-	Nodule Air-crescent	Voriconazole	Dead
Abdalla [79]/ Qatar/ AspICU	2/Putative	M/58	DM, AH, HBV	Steroid, TCZ	5	BAL/*A. niger*	-	-	-	No	Anidulafungin AMB	Dead
	Putative	M/74	No	Steroid	18	BAL/*A. terreus*	-	-	-	No	Voriconazole	Dead
Patti °° [80]/ USA/ NR	1/NR	M/73	AH	Steroid	-	BAL/*A. flavus*	Neg/-	-	-	Cavity with aspergilloma	Voriconazole	Alive
Marr [81]/ USA/ mAspICU pers.°°°	20/Probable	F/71	CPD, CKD	No	8	BAS/ *A. fumigatus*	Neg/-	-	Pos	Nodules	18 treated	Alive
	Probable	F/56	CPD, DM, AH, CVD, Obesity	No	2	-	Pos/-	-	-	Nodules		Alive
	Probable	F/70	AH	No	10	-	Pos/-	-	Pos	-		Dead
	Probable	F/68	AH, dementia	No	2	BAS/ *A. fumigatus*	Neg/-	-	-	-		Alive
	Probable	M/69	AH	No	12	BAS/*A. niger*	Neg/-	-	-	No		Dead
	Probable	M/55	AH, CVD	No	-1	BAS/ *A. fumigatus*	Neg/-	-	Neg	No		Alive
	Probable	F/85	AH, DM, CKD	No	9	BAS/ *A. fumigatus*	Pos/-	-	Pos	Nodules Cavities		Alive
	Probable	F/50	CKD, CPD	TCZ	23	BAS/ *A. fumigatus*	Neg/-	-	Pos	-		Alive
	Probable	F/45	CPD, SLE	Ocrelizumab	12	BAS/ *A. fumigatus*	Neg/-	-	Pos.	No		Alive
	Probable	F57	DM	No	4	BAS/ *A. fumigatus*	Neg/-	-	Neg	-		Alive
	Probable	M/57	Cancer	No	22	BAS/ *A. fumigatus*	Neg/-	-	Inter	-		Alive
	Probable	M/61	AH	No	16	Neg	Pos/-	-	Neg	-		Dead
	Probable	F/75	AH, CPD, DM, Obesity	No	12	BAS/ *A. fumigatus*	-	-	-	-		Alive
	Probable	F/56	CPD	TCZ	2	BAS/ *A. fumigatus*	Neg/-	-	-	-		Alive
	Probable	M70	AH	No	3	BAS/ *A. fumigatus,*	-	-	-	No		Alive

						A. niger						
	Probable	M/76	CPD	Steroid	11	BAS/ *A. fumigatus*, *A. niger*	Neg/-	-	-	-		Alive
	Probable	M/73	AH, DM	Steroid	7	BAS/ *Aspergillus*	Neg/-	-	-	No		Alive
	Probable	M/78	AH	Steroid, siltuximab	14	BAS/ *Aspergillus*	-	-	-	-		Alive
	Probable	F/56	No	TCA, anakinra	9	BAL/*A. terreus*	-	-	-	-		Alive
	Probable	M/81	DM, AH, cirrhosis	Steroid	24	BAL/*A. niger*	Neg/Pos (BAL)	-	-	Cavity		Alive
Trujillo [82]/ Spain/ EORTC	1/Probable	F/55	AH, CKD, SOT (chronic steroids)	TCZ	7	Sputum/ *A. fumigatus*	Neg/-	-	Pos	Cavity Tree-in-bud	Isavuconazole AMB	Alive
Shadrach [83]/ India/ AspICU	1/Putative	F/55	Obesity	Steroid	(post-COVID)	BAL/ *A. fumigatus*	1.2/4.7 (BAL)	-	-	Nodule	Voriconazole	Alive
Bellanger [84]/ France/ White	1/Putative	M/55	Cancer, HSCT	Steroid	19	BAL/ *A. fumigatus*	Neg/-	Neg/Pos (BAL)	-	No	AMB	Dead
Witting [85]/ USA/ NR	1/NR	M/72	No	TCZ	14	BAL/ *Aspergillus*	-/8.13 (BAL)	-	-	Cavity Fungus-ball	Voriconazole Micafungin	Alive
Haglund [86]/ Denmark/ mAspICU	1/Probable	M/52	Obesity, DM, CVD	NA	24	BAL/ *A. fumigatus* Positive DE	-/5.7 (BAL)	-/Pos (BAL)	-	-	Voriconazole	Alive
Hakamifard[87]/Iran/ ECMM	1/Probable	M/35	No	Steroid	17	BAL/ *A. ochraceus* Positive DE	2.1/-	-	-	Nodule	Voriconazole AMB	Dead
Imoto [64]/ Japan/ mAspICU	1/Probable	M/72	AH, CVD, CPD, stroke	Steroid	14	TA/ *A. fumigatus*	0.6/-	-	Pos	Nodule	Voriconazole Micafungin	Dead
Johnson [88]/ USA/ ECMM	1/Probable	M/79	AH, DM	Steroid	14	BAL/ *A. fumigatus* Positive DE	2.04/7.86 (BAL)	-	Pos	Cavity	Voriconazole	Alive
Kakamad [89]/ Iraq/ NR	1/Proven (lobectomy)	M/50	DM	NA	21	BAL/ *Aspergillus* Positive DE	-	-	-	Cavity	Voriconazole (+ surgery)	Alive
Lescure [90]/ France/ NR	1/NR	M/80	Cancer	No	-	TA/*A. flavus*	-	-	-	Cysts	Voriconazole Isavuconazole	Alive
Nasrullah [91]/ USA/ AspICU	1/Putative	M/68	AH, DM	Steroid	-	BAL/ *A. fumigatus*	Neg/2.16 (BAL)	-	Neg	No	Voriconazole	Dead
Salehi [92]/ Iran/ NR	1/Proven (Pulmonary biopsy)	M/70	No	Steroid	37	Biopsy/*A. flavus* Positive DE	-	-	-	Cavity Halo-sign	Voriconazole	Dead

Sasoni [93]/ Argentina/ mAspICU-IAPA	1/Probable	M/73	PTE	Steroid	30	Neg	1.8/-	Pos/-	-	No	Voriconazole AMB	Alive
Sharma [94]/ Austria/ NR	1/NR	F/66	AH	NA	8	TA/ *A. fumigatus*	-	-	-	No	Voriconazole	Alive
Van Someren Gréve [95]/ Netherland/ NR	1/Proven (autopsy)	M/79	AH, DM, CVD	Steroid	14	BAL/ *A. fumigatus*	1.7/7.9 (BAL)	-	-	No	Voriconazole Anidulafungin	Dead
Abolghasemi [96]/Iran/ NR	1/NR	F/66	No	Steroid	15	BAL/*A. terreus* Positive DE	4.15/-	-	-	No	Voriconazole	Dead
Steenwyk [97]/ USA/ NR	1/NR	M/57	No	-	-	BAS/ *A. fumigatus*	-	-	-	-	Caspofungin	Dead
Flikweert [98]/ Netherland/ IAPA	6/Probable	F/73	No	Steroid	19	BAL/ *A. fumigatus*	-/4.4 (BAL)	-	-	No	Voriconazole Anidulafungin	Dead
	Probable	F/58	No	Steroid	20	Neg	-/3.4 (BAL)	-	-	No	Voriconazole Anidulafungin	Dead
	Probable	M/68	No	No	1	BAL/ *A. fumigatus*	-/5.7 (BAL)	-	-	No	Voriconazole Anidulafungin	Dead
	Probable	M/78	AH, CKD	Steroid	20	Neg	-/4.3 (BAL)	-	-	No	Voriconazole Anidulafungin	Dead
	Probable	M/83	AH, DM	No	11	Neg	-/1.7 (BAL)	-	-	No	Voriconazole Anidulafungin	Dead
	Probable	M/74	No	Steroid	24	Neg	-/4.4 (BAL)	-	-	No	Voriconazole Anidulafungin	Dead
Garcìa Clemente [99]/ Spain/ NR	2/NR	F/78	Hyperparathy-roidism	No	4	BAL/ *A. fumigatus* + *A. nidulans*	-/Pos (BAL)	-	-	No	Voriconazole	Alive
	NR	M/83	AH, CVD, CKD	No	-	BAS/ *A. fumigatus*	-/Pos (BAS)	-	-	No	AMB Voriconazole	Alive
Nasri [100]/ Iran/ EORTC	1/Probable	F/42	AML	No	9	Neg	4/-	-	-	No	AMB	Dead
Martin C.S. [101]/Spain/ AspICU	3/Putative	M/71	AH	Steroid TCZ Anakinra	19	BAL/ *A. fumigatus*	-/1.4 (BAL)	-	-	No	Anidulafungin Isavuconazole	Alive
	Putative	M/73	AH, DM	Steroid	25	BAL/ *A. fumigatus*	-/1.4 (BAL)	-	-	No	Isavuconazole	Alive
	Putative	F/67	No	Steroid	27	BAL/ *A. fumigatus*	-/1.5 (BAL)	-	-	No	AMB Isavuconazole	Dead
	2/NR	40	No	Steroid	25	BAL/	-	-	-	No	Voriconazole	Dead

Alobaid [102] /Kuwait/ NR						*A. niger* Positive DE						
	NR	60	DM, AH, CVD	TCZ Steroid	26	TA/ *A. niger*	6.5/-	-	-	Cavity	AMB	Dead

Data are absolute number (percentage) unless otherwise specified.

Abbreviations. DE, direct examination of respiratory sample showing septate branching hyphae; AH, arterial hypertension; DM; diabetes mellitus; CVD, cardiovascular disease; CPD, chronic pulmonary disease; SOT, solid organ transplant; HSCT, haematopoietic stem cell transplant; CML, chronic myeloid leukaemia; MDS, myelodysplastic syndrome; CKD, chronic kidney disease; AML, acute myeloid leukaemia; ALL, acute lymphoblastic leukaemia; AS, anchylosing spondylitis; CLL, chronic lymphocytic leukaemia; PTE, pulmonary thromboembolism; HBV, hepatitis B virus; UC, ulcerative colitis; TCZ, tocilizumab; SLE, systemic lupus erythematosus; BAL, bronchial-alveolar lavage; BAS, bronchial aspirate; TA, tracheal aspirate; RS, respiratory specimen; NR, not reported; ODI, optical density index; TI, tracheal intubation; BDG, (1-3)-β-D-glucan; AMB: amphotericin B.

* Days from hospital admission to CAPA.
** Days from COVID-diagnosis to CAPA.
*** *Asp*ICU, IAPA and EORTC.
° EORTC + serology with ELISA and WesternBlot assay.
°° Case of subacute aspergillosis with fungus ball on CT scan.
°°° Cases were defined as recovery of *Aspergillus* species from respiratory fluids (tracheobronchial secretions, sputum, BAL]) or positive (index ≥1) serum or BAL markers, identified with work-up for possible secondary pneumonia, typically clinically indicated with new fever or respiratory decompensation with new focal infiltrates on chest radiograph or computed tomography scan.
^ Age is expressed as median (Inter-quartile range)
Results for a β-D-glucan assay were reported when available but did not suffice to establish case diagnosis.
