# Supplementary material for: Invasive Fungal Infections Complicating COVID-19: A Narrative Review
Source: J Fungi (Basel). 2021 Oct 29;7(11):921. doi: 10.3390/jof7110921 (PMC8620819; doi:10.3390/jof7110921)
Supplement: Supplementary file 1 [file jof-07-00921-s001.zip › tables/Table S2_CAPA studies.pdf]

## Table S2. Observational studies reporting CAPA.

| Author/ Country/ Algorithm | Cases/ total (%) | Males (%) / Median age years (IQR) | Underlying diseases (No. of patients) | Days from ICU/TI to CAPA median (IQR) | No. culture-positive samples/total (sample type) *Aspergillus* spp. (n) | No. of GM-positive samples/total (sample type)/ Median ODI | No. of PCR-positive samples/total (sample type) | No. of BDG positive samples/ total | Specific imaging findings/Type (No. of patients) | Patients treated (%)/Anti-fungal agents (No. of patients) | No. of deaths (%) |
|---|---|---|---|---|---|---|---|---|---|---|---|
| Bartoletti [20]/ Italy/ mAspICU | 30/108 (27.8) | 24 (80)/ 63 (57-70) | Obesity (10), AH (16), DM (5), CVD (6), CPD (4), SOT (1) | 4 | 19/30 (BAL) *A. fumigatus* (15) *A. niger* (3) *A. flavus* (1) | 1/30 (serum)/- 30/30 (BAL)/3.5 | 20/30 (BAL) | - | - | 16 (53.3) Voriconazole (13) Other (3) | 16 (53.3) |
| Chen [21]/ China/ NR | 1/99 (1%) | - | - | - | 1/NR *A. flavus* (1) | - | - | - | - | - | - |
| Koehler [22]/ Germany/ AspICU pers.* | 5/19 (26.3) | 3 (15.7)/ 62 (54-72) | Obesity (1), AH (3), DM (1), CPD (2) | - | 1/3 (BAL) 2/3 (TA) *A. fumigatus* (3) | 2/5 (serum)/1.7 3/3 (BAL)/2.5 | 3/3 (BAL) 1/2 (TA) | - | 5/5 Nodules (5) Cavity (1) Air-crescent (1) | 5 (100) Voriconazole (4) Isavuconazole (1) Caspofungin (2) | 3 (60) |
| Lahmer [23]/ Germany/ AspICU pers.** | 11/32 (34.3) | 7 (63.6)/ 72 (58-84) | AH (7), DM (3), CVD (2), CPD (2), CKD(1) | 4 (1-7) | 9/11 (BAL) *A. fumigatus* (9) | 4/11 (serum)/1.5 11/11 (BAL)/6.4§ | - | - | - | 11 (100) Voriconazole (5) Isavuconazole (1) Amb (5) | 4 (36.3) |
| Rutsaert [24]/ Belgium/ AspICU | 7/34 (20.5) | 7 (100)/ 66 (56-77) | Obesity (2), AH (3), DM (3), CVD (1), CKD (1), AML (1), pemphigus (1) | 8 (5-13) | 5/6 (BAL) 1/1 (TA) *A. fumigatus* (5) *A. flavus* (1) Positive DE: 4 (BAL) | 1/6 (serum)/0.8 5/6 (BAL)/2.63 | - | - | 0/7 | 6 (85.7) Voriconazole (6) Isavuconazole (2) | 4 (57.1) |
| Alanio [11]/ France/ AspICU Pers.*** | 9/27 (33.3) | 5 (71.4)/ 63 (50-76) | Obesity (3), AH (7), DM (3), CVD (2), CPD (1), myeloma (1), steroids (2) | - | 5/7 (BAL) 2/2 (BAS) *A. fumigatus* (7) | 1/9 (serum)/0.51 2/7 (BAL)/2.4 | 3/7 (BAL) 1/2 (TA) 0/8 (serum) | 4/7 | 1/9 Nodules (1) | 2 (22.2) Voriconazole (1) Caspofungin (1) | 4 (44.4) |
| Brown [25]/ UK/ IAPA | 2/62 (3.2) | 2 (100)/ 33.5 | AML (1), HSCT (1) | - | 1/2 (TA) *A. fumigatus* (1) | 0/2 (serum) 2/2 (TA) | 2/2 (TA) | 0/2 | 0/2 | 2 (100) Posaconazole (1) Itraconazole (1) Caspofungin (1) | 1 (50) |
| Van Arkel [26]/ Netherland/ mAspICU | 6/31 (19.4) | 6 (100)/ 62 (57-75) | CVD (1), CPD (1), Systemic steroids (3). | 5 (3-14) | 2/3 (BAL) 2/2 (TA) 1/1 (sputum) *A. fumigatus* (5) | - | - | - | 0/6 | 6 (100) Voriconazole (5) Anidulafungin (5) AMB (1) | 4 (66.7) |
| Nasir [27]/ Pakistan/ NR | 5/147 (3.4) | 3 (60)/ 71 (54-83) | AH (3), DM (4) | 8 (1-13) | 5/5 (TA) *A. flavus* (3) *A. niger* (1) *A. fumigatus* + *A. flavus* (1) | 0/5 (serum) | - | 2/5 | 0/5 | 5 (100) Voriconazole (3) AMB (2) | 3 (60) |

| | | | | | | | | | | | |
|---|---|---|---|---|---|---|---|---|---|---|---|
| Gangneux [10]/<br>France/<br>AspICU<br>Pers. ° | 7/45<br>(15.6) | 4 (57.1) /<br>70 (63-75) | Obesity (1),<br>AH (3), DM (2),<br>CKD (1),<br>hemopathy (1),<br>CML (1), SOT(1) | - | 6/7 (RS)<br>*A. fumigatus* (6) | 2/7 (serum) | 7/7 (RS)<br>3/7 (serum) | - | 0/7 | 7 (100)<br>Voriconazole (4)<br>Isavuconazole (3) | 2<br>(28.6) |
| Falces-Romero<br>[28]/<br>Spain/<br>AspICU-<br>EORTC | 8/5700<br>(0.1) | 6 (75)/<br>69 (61-72) | Obesity (2),<br>DM (5), CPD (2),<br>HIV (1),<br>MDS (1),<br>Haemophilia (1),<br>AS (1),<br>Golimumab (1) | 12 (7-21) | 8/8 (BAS)<br>1/1 (BAL)<br>1/1 (sputum)<br>*A. fumigatus* (9)<br>*A. nidulans* (1) | 1/2 (serum)/1.97<br>2/2 (BAL)/3 | - | - | 0/10 | 8 (100)<br>Voriconazole (5)<br>Isavuconazole (2)<br>Caspofungin (1)<br>Anidulafungin (1)<br>AMB (5) | 7<br>(87.5) |
| Zhang [29]/<br>China/<br>NR | 3/38<br>(7.9) | - | - | - | 3/NR (RS)<br>2 *Aspergillus* spp.<br>*A. fumigatus* | - | - | - | - | - | - |
| White [30]/<br>UK/<br>CAPA | 25/135<br>(18.5) | - | Obesity (6),<br>AH (8), DM (6),<br>CVD (1), CPD<br>(12), CKD (2),<br>cancer (6) | 8 (0-35) | 12/12 (BAL)<br>*A. fumigatus* (11)<br>*A. versicolor* (1) | 2/4 (serum)/2.9<br>17/19 (BAL)/5.6 | 14(14 (BAL)<br>5/5 (Serum) | 13/14 | 12/25<br>Nodules (7)<br>Cavity (5)<br>Fungus ball (1) | 18 (72)<br>Voriconazole (14)<br>Fluconazole (2)<br>Caspofungin (2)<br>Anidulafungin (2)<br>AMB (5) | 13<br>(52) |
| Ichai [31]/<br>France/<br>NR | 6/26<br>(23.1) | -/<br>64 (mean) | - | 6.5 (mean) | - | - | - | - | - | 6 (100)<br>Voriconazole (1)<br>Isavuconazole (5) | 2<br>(33.3) |
| Sarrazyn [32]/<br>Belgium/<br>AspICU | 4/131<br>(3.1) | 3 (75)/<br>75 (69-89) | Obesity (2),<br>CKD (1) | 4 | 4/4 (BAL)<br>*Aspergillus* spp. (4) | 4/4 (BAL) | 2/4 (BAL) | - | 0/4 | 3 (75)<br>Voriconazole (2)<br>AMB (1) | 4<br>(100) |
| Dupont [33]/<br>France/<br>AspICU<br>Pers. °° | 19/106<br>(17.9) | 15 (79)/<br>69 (62-73) | Obesity (1),<br>AH (7), DM (7),<br>CVD (1), CPD<br>(8), cancer (4),<br>CKD (2), HIV<br>(1), HBV (1) | 10 (7-15) | 7/8 (BAL)<br>6/6 (BAS)<br>3/4 (TA)<br>*A. fumigatus* (14)<br>*A. niger* (1)<br>*A. calidoustus* (1)<br><br>Positive DE:<br>2 (BAL) + 1 (TA) | 5/9 (BAL)/1.57 | - | - | 3/19<br>Nodules (2)<br>Cavity (2) | 9 (47.3)<br>Voriconazole (9)<br>Caspofungin (1) | 7<br>(36.8) |
| Mitaka [34]/<br>USA/<br>AspICU | 4/7<br>(57.1) | 4 (100)/<br>78 (77-80) | AH (1), DM (1),<br>CVD (2),CPD<br>(1), cancer (2) | 10 (8-12) | 4/4 (BAL)<br>*A. fumigatus* (4) | 1/4 (serum) | - | - | 1/4<br>Cavity (1)<br>Air-crescent (1) | 4 (100)<br>Voriconazole (3)<br>Caspofungin (1) | 4<br>(100) |
| Borman [35]/<br>UK/<br>AspICU<br>Pers. °°° | 15/61<br>(24.6) | 9 (60)/<br>57 (48-66) | - | - | 2/4 (BAL)<br>2/3 (TA)<br>1/1 (sputum)<br>*A. fumigatus* (5)<br><br>Positive DE: 1 (BAL) | 5/15 (serum)/1.7<br>4/5 (BAL)/5.2 | 2/3 (BAL)<br>1/8 (serum) | 12/15 | - | - | - |

| | | | | | | | | | | | |
|---|---|---|---|---|---|---|---|---|---|---|---|
| Rothe [36]/<br>Germany/<br>NR | 9/140<br>(6.4) | - | - | - | - | - | - | - | - | - | - |
| Wang [37]/<br>China/<br>EORTC | 8/104<br>(7.7) | 8 (100)/<br>73 (mean) | AH (7), DM (2),<br>CVD (1), CPD<br>(2), CKD (2) | 19 (mean) | 4/NR (BAL)<br>4/NR (sputum)<br>*A. fumigatus* (8) | - | - | - | 2/8<br>Nodules (1)<br>Cavity (1) | - | - |
| Chauvet [38]/<br>France/<br>Multiple $ | 6/46<br>(13) | 6 (100)/<br>68 (63-70) | Obesity (1),<br>AH (3), DM (1),<br>CVD (3), CPD<br>(3), CKD (2),<br>vasculitis (1),<br>SOT (1) | 7 (6-14) | 2/2 (BAL)<br>2/2 (TA)<br>*A. fumigatus* (4) | 2/3 (BAL) | 1/1 (TA) | - | 1/6<br>Cavity (1) | 5 (83.3)<br>Voriconazole (2)<br>Isavuconazole (1)<br>Caspofungin (1)<br>AMB (2) | 4<br>(66.7) |
| Machado [39]/<br>Spain/AspICU | 8/239<br>(3.3) | 6 (75)/<br>64 (61-73) | Obesity (4),<br>AH (7), DM (1),<br>CPD (3), CKD<br>(2), CLL (1), SOT<br>(1) | 15 (10-19) | 5/5 (BAS)<br>2/2 (BAL)<br>1/1 (TA)<br>*A. fumigatus* (5)<br>*A. lentulus* (1)<br>*A. citrinoterreus* (1)<br>*A. fumigatus* +<br>*A. terreus* +<br>*A. awamori* (1) | 4/8 (serum)/1.25<br>2/8 (BAL)/4.9 | - | 2/8 | 1/8<br>Nodules (1) | 5 (62.5)<br>Voriconazole (2)<br>Isavuconazole (4)<br>AMB (2) | 8<br>(100) |
| Segrelles-Calvo<br>[40]/<br>Spain/<br>EORTC | 7/215<br>(3.3) | 5 (71.4)/<br>58 (55-70) | Obesity (2),<br>AH (4), DM (3),<br>CPD (1), allergic<br>alveolitis (1),<br>cancer (1) | 12<br>(10-15) | 7/7 (RS)<br>6 BAL, 2 BAS, 1<br>sputum<br>*A. fumigatus* (3)<br>*A. flavus* (2)<br>*A. niger* (2) | - | - | - | - | 5 (71.4)<br>Itrraconazole (3)<br>AMB (1) | 5<br>(71.4) |
| Roman-Montes<br>[41]/<br>Mexico/<br>IAPA pers. ^ | 14/144<br>(9.7) | 11 (78.6)/<br>48 (mean) | Obesity (12),<br>AH (4),<br>DM (4), HIV (1) | 9 (3-13) | 9/14 (TA)<br>*Aspergillus* spp. (1)<br>*A. fumigatus* (5)<br>*A. flavus* (1)<br>*A. niger* (1)<br>*A. versicolor* (1) | 6/9 (serum)/1.42<br>8/14 (TA)/5.8 | - | - | 0/14 | 12 (85.7)<br>Voriconazole (11)<br>Caspofungin (1) | 8<br>(57.1) |
| Permpalung<br>[42]/<br>USA/<br>Personal ^^ | 39/396<br>(9.8) | 22 (56.4)/<br>66 (55-70) | Obesity (16), AH<br>(29), DM (30),<br>CVD (18), CPD<br>(16), CKD (17),<br>liver disease<br>(14), HIV (1),<br>cancer (13) | 12 (3-22) | 13/39<br>(2 BAL, 11 RS)<br>*Aspergillus* spp. (13) | 8/39 (serum)<br>2/39 (BAL) | - | 17/39 | 9/39<br>Nodules (9) | 19 (48.7) | 22<br>(56.4) |
| Delliere [43]/<br>France/<br>mAspICU-<br>EORTC | 21/366<br>(5.7) | 16 (76.1)/<br>63 (57-68) | Obesity (4),<br>AH (14), DM (9),<br>CVD (2), CPD<br>(2), myeloma (1),<br>SOT (1), steroids<br>(3) | 6 (1-15) | 19/21 (RS)<br><br>Positive DE: 2 (RS) | 5/20 (serum)<br>3/14 (BAL) | 2/16 (serum)<br>10/12 (RS) | 9/20 | - | 7 (33.3) | 15<br>(71.4) |
| Falcone [44]/<br>Italy/NR | 1/315<br>(0.3) | - | - | - | - | - | - | - | - | - | - |

| | | | | | | | | | | | |
|---|---|---|---|---|---|---|---|---|---|---|---|
| Fekkar [45]/<br>France/<br>EORTC | 6/260<br>(2.3) | 5 (83.3)/<br>56 (49-72) | Obesity (5),<br>AH (5), DM (1),<br>SOT (3) | 10 (6-42) | 3/4 (BAL)<br>2/2 (TA)<br>*A. fumigatus* (3) | 1/6 (serum)/1.19<br>2/5 (BAL)/1.5 | 2/2 (TA)<br>2/2 (BAL)<br>2/6 (Serum) | 2/6 | - | 5 (83.3)<br>Voriconazole (5)<br>Isavuconazole (5)<br>Caspofungin (5)<br>AMB 82) | 4<br>(66.7) |
| Gouzien [46]/<br>France/<br>mAspICU | 2/53<br>(3.8) | - | - | - | 2/53 *Aspergillus* spp.<br>(1 BAL, 1 TA) | 1/53 (serum) | - | - | - | 1 | - |
| Van Grootveld<br>[47]/<br>Netherland/<br>ECMM | 11/63<br>(17.5) | 14 (73.7)/<br>65 (59-72) | AH (6),<br>DM (5), CVD (3),<br>CPD (7), PT (2),<br>cancer (1) | 17<br>(14-21)& | 3/11 (BAL)<br>4/8 (TA)<br>*A. fumigatus* (7)<br><br>Positive DE: 1 (BAL) | 10/11 (BAL)/4.4 | 6/11 (BAL)<br>7/8 (BAS) | - | - | 9 (47.4)<br>AMB (9)<br>Voriconazole (5) | 10<br>(52.6) |
| Maes [48]/<br>UK/<br>IAPA | 3/94<br>(3.2) | - | - | - | 0/3 (BAL) | 3/3 (BAL)/1 | 1/3 (BAL) | - | - | 3 (100)<br>AMB (3) | 2<br>(66.7) |
| Meijer [49]/<br>Netherland/<br>ECMM | 13/66<br>(19.7) | 10 (76.9)/<br>68 (62-74) | AH (2),<br>DM (3), CVD (7),<br>CPD (1), UC (2),<br>CKD (1) | - | 8/8 (BAL)<br>5/5 (TA)<br>*A. fumigatus* (13) | 0/6 (serum)<br>4/5 (TA) + 2/12<br>(BAL)/4.5 | 4/5 (BAL)<br>2/2 (TA) | 1/1 | 2/13<br>Nodules (1)<br>Cavity (1) | 13 (100)<br>Voriconazole (13)<br>Caspofungin (5)<br>AMB (5) | 6<br>(46.1) |
| Van Biesen<br>[50]/<br>Netherland/<br>Personal $$$ | 9/42<br>(21.4) | 6 (66.7)/<br>73 (65-75) | Obesity (8),<br>AH (3), DM (1),<br>CPD (4), SOT<br>(1), chronic<br>steroid (1) | 3 (1-5) | 7/9 (BAL)<br>*A. fumigatus* (5)<br>*A. flavus* (1)<br>*A.* terreus (1) | 9/9 (BAL)/4 | - | - | 0/9 | 9 (100)<br>Voriconazole (9)<br>AMB (9) | 2<br>(22.2) |
| Obata [51]/<br>USA/<br>NR | 4/226<br>(1.8) | - | - | - | - | - | - | - | - | - | - |
| Razazi [52]/<br>France/<br>mAspICU-<br>IAPA | 7/90<br>(7.8) | - | - | 6 (5-11) | 4/24 (BAL)<br>*Aspergillus* spp. (4)<br><br>Positive DE: 2 (BAL) | 5/88 (serum) | - | 9/88 | - | - | - |
| Ripa [2]/<br>Italy/AspICU<br>Pers. ^^^ | 11/731<br>(1.5) | - | - | - | 8/NR (4 BAL, 4 BAS)<br>*A. fumigatus* (7)<br>*A.* terreus (1) | 6/NR (BAL) | - | - | - | - | - |
| Pintado [53]/<br>Mexico/<br>ECMM | 16/83<br>(19.3) | 11 (68.8)/<br>64 (mean) | Obesity (6),<br>AH (4), DM (5),<br>CPD (1),<br>cancer (3) | 6 (4-9) | 2/16 (BAL)<br>*A. fumigatus* (2) | 7/16 (serum)<br>9/16 (BAL) | - | - | - | - | 5<br>(31.3) |
| Versych [54]/<br>France/<br>IAPA | 2/54<br>(3.7) | 2 (100)/<br>63.5 | AH (1), DM (1) | 6.5 | 1/1 (BAL)<br>1/1 (TA)<br>*A. fumigatus* (2)<br><br>Negative DE:<br>1 (BAL) | 2/2 (serum)/2.34<br>1/2 (BAL)/2.46 | - | 2/2 | 2/2<br>Cavity (2)<br>Nodules (1)<br>Halo sign (1) | 2 (100)<br>Voriconazole (2) | 2<br>(100) |

| | | | | | | | | | | | |
|---|---|---|---|---|---|---|---|---|---|---|---|
| Reizine [55]/<br>France/<br>ECMM | 10/49<br>(20.4) | 6 (60)/<br>72 (57-77) | Obesity (1), DM<br>(2), CKD (1),<br>CML (1), SOT<br>(1) | 6 (3-12) | 8/10 (BAS)<br>1/10 (sputum)<br>*A. fumigatus* (9) | 3/10 (serum)<br>4/10 (BAS) | 9/10 (BAS) | - | Wedge-cons. (7)<br>Halo sign (1) | 8 (80)<br>Voriconazole (6)<br>Isavuconazole (4) | 4<br>(40) |
| Oliva [56]/<br>Italy/<br>mAspICU-<br>IAPA | 2/55<br>(3.6) | 2 (100)/<br>48.5 | Obesity (1) | - | 0/2 (RS) | 2/2 (RS)/2.3 | - | - | 0/2 | - | 0 |
| Campochiaro<br>[57]/Italy/<br>NR | 1/65<br>(1.5) | 1 (100)/<br>68 | - | - | 1/1 (BAL)<br>*A.* terreus (1) | - | - | - | - | 1 (100)<br>Voriconazole (1) | 1<br>(100) |
| Wasylyshyn<br>[58]/<br>USA/ECMM | 3/256<br>(1.2) | 2 (66.7)/<br>67 | Obesity (2),<br>AH (2), DM (1),<br>epilepsy (1) | 13 §§ | 2/3 (TA)<br>*A. fumigatus* (2) | 1/2 (serum)/3.1<br>1/1 (BAL)/1.4 | - | - | 2/3<br>Cavity (2) | 2 (66.7)<br>Voriconazole (2)<br>Isavuconazole (1)<br>Micafungin (1)<br>AMB (1) | 1<br>(33.3) |
| Nebreda-<br>Mayoral [59]/<br>Spain/<br>NR | 3/712<br>(0.4) | - | - | - | 3/3 (BAS)<br>*A. fumigatus* (2)<br>*A. niger* (1) | - | - | - | - | 3 (100)<br>Voriconazole (2)<br>Isavuconazole (2) | 2<br>(66.7) |
| Signorini [60]/<br>Italy/<br>EORTC | 2/92<br>(2.2) | - | - | - | 2/NR<br>*Aspergillus* spp. (2) | - | - | - | - | - | - |
| Rabagliati [61]/<br>Chile/<br>ECMM | 14/146<br>(9.6) | 8 (57.1)/<br>65 (55-73) | Obesity (3),<br>AH (7),<br>DM (2),<br>CPD (4) | 15 (10-23) | 7/14 (TA)<br>*A. niger* (4)<br>*A. fumigatus* (3)<br>*A.* terreus (1)<br>*A. lentulus* (1) | 6/14 (serum)/1.2<br>2/14 (BAL)/4.6 | - | - | 1/13<br>Cavity (1) | 11 (78.6)<br>Voriconazole (10)<br>AMB (1) | 4<br>(28.6) |
| Lamoth [62]/<br>Switzerland/<br>mAspICU<br>pers. $$ | 3/118<br>(2.5) | 3 (100)/<br>62 (mean) | Obesity (2), A<br>H (2), DM (1),<br>CPD (1) | 7 (3-8) | 3/3 (TA)<br>*A. fumigatus* (3) | 1/3 (serum)/0.73 | 1/3 (TA) | 1/3 | 0/3 | 3 (100)<br>Voriconazole (3) | 1 (33.3) |
| Yang [63]/<br>China/<br>NR | **2/52<br>(3.8)** | **-** | **-** | **-** | **2/NR (RS)<br>*A. fumigatus* (1)<br>*A. flavus* (1)** | **-** | **-** | **-** | **-** | **-** | - |

Data are absolute number (percentage) unless otherwise specified.

**Abbreviations**. DE, direct examination of respiratory sample showing septate branching hyphae; AH, arterial hypertension; DM, diabetes mellitus; CVD, cardiovascular disease; CPD, chronic pulmonary disease; SOT, solid organ transplant; ICU, intensive care unit; IQR, interquartile range; TI, tracheal intubation; ODI, optical density index; BDG, (1,3)-beta-D-glucan; BAL, bronchial alveolar lavage; TA, tracheal aspirate; BAS, bronchial aspirate; CKD, chronic kidney disease; AML, acute myeloid leukaemia; HSCT, haematopoietic stem cell transplantation; CML, chronic myeloid leukaemia; HIV; human immunodeficiency virus; MDS, myelodysplastic syndrome; AS, ankylosing spondylitis; CLL, chronic lymphocytic leukaemia; PT, pulmonary thromboembolism; UC, ulcerative colitis; RS, respiratory specimen, AMB, amphotericin B.

* Modified AspICU algorithm with the addition that positive galactomannan GM (≥1) from BAL or TA, or two consecutively but separately drawn positive serum samples (≥1) were accepted as entry criterion.

** Putative IPA was assumed in one of the following conditions: cultural growth of *Aspergillus* spp from BAL specimens.; GM optical density index (ODI) >0.5 in serum, GM ODI >1 in respiratory tract specimen from BAL. Clinical signs and radiological signs were in line with the modified AspICU score and CAPA definitions.
*** Putative IPA was considered if *Aspergillus* spp were identified in BAL culture; or if two of the following conditions were met (ie, presence of Aspergillus spp in bronchial aspiration (BAS) culture; positive *Aspergillus fumigatus* qPCR in BAL, BA, or serum; galactomannan ODI >0.8 in BAL; GM ODI >0.5 in serum; and β-D-glucan >80 pg/mL in serum).
° putative IA: AspICU algorithm which includes PCR together with culture; probable IA: putative IA + one positive blood biomarker (GM and/or PCR).
°° AspICU algorithm plus BAL GM results; COVID-19 considered as a risk factor.
°°° modified AspICU algorithm incorporating PCR, serology, and angioinvasion biomarkers as proposed by Gangneux.
^ modified AspICU algorithm by Schauwvlieghe, considering a positive serum GM or Aspergillus spp. growth from a TA, since bronchoscopies were not performed at Authors' institution due to safety concerns for healthcare workers.
^^ Probable CAPA: presence of new cavitary lung lesion(s) on chest CT without alternative explanation, positive serum GM EIA index ≥0.5, positive BAL GM index ≥1.0, or positive culture for *Aspergillus* species in BAL. Possible CAPA: positive BAL GM index 0.5 – 1.0; positive serum BDG ≥80 pg/ml without alternative explanation; or culture with growth of *Aspergillus* species in TA.
^^^ AspICU algorithm plus serum or BAL GM index.
$ AspICU by Blot, IAPA by Schauwvlieghe and EORTC algorithm.
$$ mAspICU by Verweij. Results of BAS cultures were also considered for defining a putative IPA category (*Aspergillus* spp. recovered from culture of ≥2 consecutive BAS in the absence of bronchoscopy and BAL sample).
$$$ AspICU algorithm without the host risk factor+ BAL GM index.
§ In CAPA non-survivors; median was 4.6 days in CAPA-survivors.
§§ Median days since COVID-diagnosis to CAPA.
& Median days since symptoms onset to CAPA.
