# Supplementary material for: Invasive Fungal Infections Complicating COVID-19: A Narrative Review
Source: J Fungi (Basel). 2021 Oct 29;7(11):921. doi: 10.3390/jof7110921 (PMC8620819; doi:10.3390/jof7110921)
Supplement: Supplementary file 1 [file jof-07-00921-s001.zip › tables/Table S3_CAPA diagnostic criteria.pdf]

Table S3. Diagnostic algorithms used for CAPA.

Aspergillosis type	AspICU (Blot et al., July 2012)	Modified AspICU^ IAPA in ICU (Schauwvlieghe et al., July 2018)	EORTC/MSG (Donnelly et al., December 2019)	Modified AspICU^ IAPA in ICU (Verweij et al., June 2020)	CAPA (White et al., August 2020)	ECMM/ISHAM CAPA (Koehler, et al., December 2020)
**Proven**	Positive histopathology, cytopathologic or direct examination on sterile material (hyphae + tissue damage).<br><br>Positive culture on sterile material.	NS	Histopatohologic, cytopathologic, or direct microscopic examination of a specimen obtai ed by needle aspiration or biopsy in which hyphae or melanized yeast-like forms are seen accompanied by evidence of associated tissue damage<br>Recovery of a hyaline or pigmented mold by culture of a specimen obtained by a sterile procedure from a normally sterile and clinically or radiologically abnormal site consistent with an infectious disease process.<br>Amplification of pungal SNA by PCR combined with DNA sequencing whne molds are seen in formalin-fixed or paraffin-embedded tissues.	Positive histopathology and *Aspergillus* growth on culture or positive *Aspergillus* PCR in tissue	Histology/Microscopy demonstrating dichotomous septate hyphae in tissue.<br><br>Positive culture from tissue.	At least **one** of the following:<br>Histopathological or direct microscopic detection of fungal hyphae, showing invasive growth with associated tissue damage<br>**or**<br>Positive culture, microscopy, histology or PCR from oulmonary site obtained by a sterile aspiration or biopsy
**Putative**	**All 4 criteria must be met**<br>1. *Aspergillus*-positive LRT specimen culture (entry criterion)<br>2. Compatible signs and symptoms**<br>3. Abnormal thoracic CT or X-ray imaging<br>4a. Host risk factors***<br>**or**<br>4b. Semi-quantitative *Aspergillus*-positive culture of BAL fluid + positive direct microscopy showing branching hyphae without bacterial growth	**Clinical criteria**<br>(One of these sign/symptoms**)<br><br>**Radiological criteria**<br>Any infiltrate on pulmonary imaging by CXR or CT scan.<br>**Mycological criteria**<br>One of the following must be present<br>Histopathology or direct microscopic evidence of dichotomous hypahe with positive culture for *Aspergillus* from tissue<br>**Or**<br>Positive BAL culture<br>**Or**<br>BAL GM index ≥ 1<br>**Or**<br>Serum GM index ≥ 0.5	NA	NA	**Clinical criteria**<br>(One of these sign/symptoms**)<br><br>**If non-specific radiology**<br><u>>2 of:</u><br>1. Positive culture of NBL/BAL<br>2. Positive GM in NBL/BAL (>1.0)<br>3. Positive GM in serum (>0.5)<br>4. Positive *Aspergillus* PCR in BALF or blood<br>5. Positive 1-3-β-D-glucan in serum/plasma<br><br>**If radiological findings typical of IA***<br><u>1 of the above criteria</u>	NA

**Probable**	NA	NA	**Host factors**<br>-Neutropenia >10 d<br>-Hematologic malignancy<br>-aHSCT/SOT<br>-Corticosteroids at a dose of ≥0.3 mg/kg for ≥3 weeks in the past 60 days<br>-Therapy with T/B-cell immunosuppressants, during the past 90 days<br>-Inherited severe immunodeficiency<br>-Acute GvHD grade III or IV.<br><br>**Clinical features**<br>One of these 4 pattern in chest-CT scans:<br>1. Dense, well-circumscribed lesions(s) with or without a halo sign<br>2. Air crescent sign<br>3. Cavity<br>4. Wedge-shaped and segmental or lobar consolidation<br><br>**Mycological evidence**<br>One of the following:<br>1. Microscopical detection of fungal elements in sputum, BAL, bronchial brush, or aspirate indicating a mold<br>2. Culture from sputum/BAL/BAS/TA, brushing<br>3. Serum GM ODI > 1<br>4. BAL GM > 1<br>5. Serum GM > 0.7 + BAL GM > 0.8<br>6. Two consecutive positive plasma PCR<br>7. Two positive BAL PCR<br>8. Positive plasma PCR + positive BAL PCR.	**A:** Pulmonary infiltrate and at least one of the following:<br>Serum GM index > 0.5<br>**or**<br>BAL GM index ≥ 1<br>**or**<br>Positive BAL culture<br><br>**B:** Cavitating infiltrate and at least one of the following:<br>Positive sputum culture<br>**or**<br>Positive TA culture	NA	**Host factor**<br>Patient with COVID-19 needing intensive care and a temporal relationship (entry criterion)<br><br>**Clinical factors**<br>Pulmonary infiltrates, preferably documented by chest CT, or cavitating infiltrate (not attributed to another cause)<br><br>**Mycological evidence**<br>At least one of the following:<br>Microscopic detection of fungal elements in bronchoalveolar lavage, indicating a mould;<br>**or**<br>positive BAL culture;<br>**or**<br>serum GM index > 0.5 or serum LFA index > 0.5<br>**or**<br>BAL GM index ≥ 1 **or** BAL LFA index ≥ 1<br>**or**<br>two or more positive aspergillus PCR tests in plasma, serum, or whole blood;<br>**or**<br>a single positive aspergillus PCR in BAL fluid (<36 cycles);<br>**or**

						a single positive aspergillus PCR in plasma, serum, or whole blood, and a single positive in BAL fluid (any threshold cycle permitted).
**Possible**	NA		Probable aspergillosis, without mycological criteria.		NA	**Host factor**<br>Patient with COVID-19 needing intensive care and a temporal relationship (entry criterion)<br><br>**Clinical factors**<br>Pulmonary infiltrates, preferably documented by chest CT, or cavitating infiltrate (not attributed to another cause)<br><br>**Mycological evidence**<br>At least one of the following: microscopic detection of fungal elements in non-BAL indicating a mould<br>**or**<br>positive non-BAL culture<br>**or**<br>single non-BAL GM index >4.5<br>**or**<br>non-BAL GM index >1.2 twice or more<br>**or**<br>non-BAL GM index >1 plus another non-BAL mycology test positive (non-BAL PCR or LFA)

**Colonisation**	When ≥ 1 criterion for putative IPA is not met	NA	NA	NA	NA	NA
**Comments**	Biomarkers (GM, 1,3-β-D-glucan) not considered for diagnosis.	Serum and/or BAL GM added to case definition; no host risk factors among the diagnostic criteria	In the last update, PCR was included. Host risk factor necessary, poor applicability to ICU patients. CT scan required for radiological criteria.	Serum and/or BAL GM added to case definition; traditional EORTC risk factors are not considered.	The number of required mycological criteria change depending on radiological findings ("non-specific" *vs* "typical"); serum 1,3-β-D-glucan included as a mycological criterion.	Entry criterion: COVID-19 + ICU admission + temporal relationship with IPA.<br><br>Includes biomarkers testing on non-BAL samples.

Abbreviations. NA: not applicable; NS, not specified; IPA, invasive pulmonary aspergillosis; IAPA, influenza-associated pulmonary aspergillosis; ECMM/ISHAM, European Confederation for Medical Mycology and the International Society for Human and Animal Mycology; CAPA, covid-associated pulmonary aspergillosis; PCR: polymerase chain reaction; GM: galactomannan antigen; BAL: bronchoalveolar lavage; BAS; bronchial spirate; TA, tracheal aspirate; LRT, low respiratory tract; CXR, chest X-ray; CT, computed tomography; LFA, lateral-flow assay; GM; galactomannan; aHSCT, allogenic haematopoietic stem cell transplant; SOT, solid organ transplant; GvHD, graft versus host disease.

^ These algorithms were originally developed for diagnosing influenza-associated pulmonary aspergillosis. During the COVID-19 pandemic, IAPA algorithms were used to diagnoise CAPA.
* Nodules, haloes, cavities, wedge-shapes, and segmental or lobar consolidation.
** Fever refractory to at least 3 days of appropriate antibiotic therapy, recrudescent fever after a period of defervesce of at least 48 hours while still on antibiotics and without other apparent cause, pleuritic chest pain, pleuritic rub, dyspnoea, haemoptysis, worsening respiratory insufficiency in spite of appropriate antibiotic therapy and ventilatory support.
*** Neutropenia (Neutrophil < 500 mm^3) preceding or a the time of ICU admission, haematological or oncological malignancy treated with cytotoxic agents, glucocorticoid treatment (prednisone equivalent > 20 mg/d), congenital or acquired immunodeficiency.
