# Supplementary material for: Invasive Fungal Infections Complicating COVID-19: A Narrative Review
Source: J Fungi (Basel). 2021 Oct 29;7(11):921. doi: 10.3390/jof7110921 (PMC8620819; doi:10.3390/jof7110921)
Supplement: Supplementary file 1 [file jof-07-00921-s001.zip › tables/Table S4 S5_Candida reports and studies.pdf]

Table S4. Case report/series describing candidemia and yeast fungemia associated with COVID-19.

Author/ Country	Cases	Sex/ Age	Underlying conditions	Steroid/ Target therapies COVID-19	Days from ICU/TI to Candidemia (IQR)	CVC	TPN	ATB	IMV	*Candida* species	Secondary focus	Antifungals	Outcome
Al-Hatmi [108]/ Oman	5	M/76	No	No	-	Yes	No	Yes	Yes	*C. albicans*	No	No	Dead
		M/68	AH	No	-	Yes	No	Yes	Yes	*C. albicans*	No	Yes (Caspofungin 2, Caspo + AMB 1, Caspo + Vor 1)	Dead
		M/38	AH, CVD	No	-	Yes	No	Yes	Yes	*C. albicans*	No	Yes	Dead
		M/64	AH	No	-	Yes	No	Yes	Yes	*C. albicans*, *C. tropicalis*	No	Yes	Alive
		M/49	No	No	-	Yes	No	Yes	Yes	*C. albicans*	No	Yes	Alive
Antinori [109]/ Italy	3	M/67	CVD	TCZ	13	Yes	Yes	Yes	Yes	*C. albicans*	Endophtalmitis, endocarditis	Caspofungin Fluconazole	Alive
		M/58	AH	TCZ	17	No	Yes	No	No	*C. tropicalis*	No	Caspofungin	Alive
		M/78	DM, Obesity	TCZ	14	Yes	Yes	Yes	No	*C. parapsilosis*	No	Caspofungin Fluconazole	Alive
Khatib [110]/ Qatar	1	M/60	Obesity, AH, DM, CVD	Steroids TCZ	30	Yes	Yes	Yes	Yes	*C. parapsilosis*	No	Anidulafungin	Dead
Alataby [111]/ USA	1	M/67	Obesity, AH, DM, CPD, CKD, HCV, plegia	Steroids BAR	-	Yes	No	Yes	Yes	*C. dubliniensis*	No	Micafungin Fluconazole	Alive
Allaw [112]/ Lebanon	2	M/75	Cancer	Yes	40	Yes	-	Yes	Yes	*C. auris*	No	Yes	Alive
		M/66	Cancer	Steroids	62	Yes	-	Yes	Yes	*C. auris*	No	Yes	Dead
Bhagali [113]/ India	1	M/42	AH, DM	Steroids TCZ	-	-	-	-	Yes	*C. albicans*	Retinitis	Yes	Alive
Nobrega d.A. [114]/ Brazil	3	F/74	DM, AH, CKD, CVD	-	34	Yes	No	Yes	Yes	*C. auris*	No	Anidulafungin	Dead
		M/68	Obesity, DM, CKD	-	8	Yes	No	Yes	Yes	*C. auris*	No	Anidulafungin	Dead
		F/88	AH, DM, dementia	-	11	Yes	No	Yes	Yes	*C. auris*	No	Anidulafungin	Dead
Riche [115]/ Brazil	11	M/44	DM, psoriasis, schizophrenia	Steroids	-	Yes	-	Yes	8 total	*C. albicans*	No	Anidulafungin	Alive
		F/71	AH, CVD	Steroids	1	Yes	-	Yes		*C. glabrata*	No	No	Dead
		F/69	DM, AH, obesity, CVD, CPD	Steroids	9	Yes	-	Yes		*C. albicans*	No	Anidulafungin	Dead
		F/37	CPD	Steroids	8	Yes	-	Yes		*C. tropicalis*	No	AMB Fluconazole	Dead
		F/87	AH, CVD	Steroids	6	Yes	-	Yes		*C. albicans*	No	Anidulafungin	Dead
		M/66	AH	Steroids	14	Yes	-	Yes		*C. albicans*	No	Anidulafungin	Alive
		M/91	CPD	Steroids	7	Yes	-	Yes		*C. albicans*	No	Anidulafungin	Dead

		M/54	DM, AH, obesity, Raynaud	Steroids	0	No	-	Yes		*C. albicans*	No	Fluconazole	Alive
		M/41	HIV, HCV, CVD	Steroids	0	Yes	-	No		*C. albicans*	No	Fluconazole	Dead
		M/57	DM, AH	Steroids	22	Yes	-	Yes		*C. albicans*	No	Voriconazole Anidulafungin	Dead
		M/59	AH, CKD	Steroids	3	Yes	-	Yes		*C. albicans*	No	Fluconazole	Dead
Sari [116]/ Indonesia	1	M/54	AH, DM	Steroids TCZ	-	-	-	-	No	*C. tropicalis*	No	Micafungin	Alive
Tafti [117]/ USA	1	M/54	Cirrhosis, HBV, cancer	-	-	-	-	-	No	*C. albicans*	-	-	-
Ventoulis [118]/ Greece	2	M/76	AH	-	35	Yes	Yes	Yes	Yes	*S. cerevisiae*	No	Anidulafungin Fluconazole	Alive
		M/73	AH, DM	-	15	Yes	Yes	Yes	Yes	*S. cerevisiae*	No	Anidulafungin Fluconazole	Alive
Villanueava [119]/ Mexico	6	M/51	AH, DM, obesity	Steroids BARI	37	Yes	-	Yes	Yes	*C. auris*	No	Caspofungin Anidulafungin	Dead
		M/55	AH, DM, CVD	Steroids BARI	29	Yes	-	Yes	Yes	*C. auris* *C. glabrata*	No	Anidulafungin	Dead
		M/64	-	Steroids BARI	13	Yes	-	Yes	Yes	*C. auris*	No	Caspofungin Voriconazole AMB	Dead
		M/64	AH, obesity	Steroids TCZ BARI	10	Yes	-	Yes	Yes	*C. auris* *C. glabrata*	No	Anidulafungin Isavuconazole	Dead
		F/54	AH, obesity	Steroids BARI	31	Yes	-	Yes	Yes	*C. auris*	No	AMB Caspofungin Voriconazole	Dead
		M/46	Obesity	Steroids BARI	27	Yes	-	Yes	Yes	*C. auris*	No	Voriconazole Caspofungin	Dead
Al-Argan [120]/ Saudi Arabia	1	M/70	DM	Steroids	-	-	-	Yes	Yes	*C. albicans*	No	Caspofungin Anidulafungin	Alive
Gorkem [121]/ Turkey	1	M/81	AH, cancer	-	6	Yes	Yes	Yes	Yes	*C. albicans*	No	No	Dead
Awada [122]/ Lebanon	1	M/34	No	-	-	Yes	No	Yes	Yes	*C. duobushaemulonii*	No	Caspofungin Fluconazole Flucytosine	Dead
Gorospe-Sarasua [123]/ Spain	1	M/53	No	TCZ	-	Yes	No	Yes	Yes	*C. albicans* (Blood culture and vertebral biopsy)	Costochondritis and spondylitis (4 months later)	Fluconazole	Alive

Data are number (percentage), unless otherwise specified.

Abbreviations. AH, arterial hypertension; DM, diabetes mellitus; CVD, cardiovascular disease; CPD, chronic pulmonary disease; SOT, solid organ transplant; ICU, intensive care unit; IQR, interquartile range; TI, tracheal intubation; CVC, central venous catheter; TPN, total parenteral nutrition; ATB, broad-spectrum antibiotics; IMV, invasive mechanical ventilation; CKD, chronic kindeny disease; AMB, amphotericin B; BARI, baricitinib; TCZ, tocilizumab; NS, not specified.

Table S5. Observational studies reporting candidemia and yeast fungemia associated with COVID-19.

Author/ Country	No. of cases/ total (%)	Males (%)/ Median age years (IQR)	Underlying diseases (No.)	Steroid / Biological therapies for COVID-19 (No. of pts)	Days from ICU/TI to Candidemia	No. of patients with CVC (%)	No. of patients with TPN (%)	No. of patients received ATB (%)	No. of patients under IMV (%)	No. of *Candida* species (%)	Patients treated (%)/Anti-fungal agents used (No. of patiets)	No. of deaths (%)
Chowdahary [124]/ India	15/596 (2.5)	11 (73.3)/ 67 (52-72)	Obesity (1), AH (7), DM (6), CVD (3), CKD (3), liver disease (3)	Steroids (10)/ TCZ (3)	12 (8-14)	15 (100)	-	15 (100)	8 (53.3)	*C. albicans* (3)<br>C: *tropicalis* (1)<br>*C. krusei* (1)<br>*C. auris* (10)	15 (100)<br>Micafungin (13)<br>L-AMB (6)	8 (53.3)
Chen [21]/ China	4/99 (4)	-	-	-	-	-	-	-		*C. albicans* (3)<br>*C. glabrata* (1)	-	-
Bishburg/ USA	8/89 (8.9)	4 (50)/ 63 (44-73)	Polimialgia reumatica (1)	Steroids (8)/ TCZ (4)	26 (21-35)	8 (100)	-	-	8 (100)	*C. albicans* (2)<br>*C. glabrata* (2)<br>*C. tropicalis* (2)<br>*C. parapsilosis* (2)	8 (100)<br>Caspofungin (5)<br>Fluconazole (7)	3 (37.5)
Hughes [126]/ UK	3/836 (0.4)	-	-	-	-	-	-	-	-	*C. albicans* (3)	-	-
Zhang [137]/ China	5/38 (13.2)	-	-	-	-	-	-	-	-	*Candida* spp. (4)<br>*T. asahii* (1)	-	-
White [30]/ UK	5/135 (3.7)	-	Obesity (1), CVD (1), CPD (1), cancer (1)	-	-	5 (100)	-	-	5 (100)	*C. albicans* (5)<br>C: *parapsilosis* (1)	4 (80)<br>Caspofungin (2)<br>L-AMB (2)<br>Fluconazole (4)	3 (60)
Rothe [36]/ Germany	3/140 (2.1)	-	-	-	-	-	-	-	-	*C. albicans* (2)	-	-
Posteraro [127]/ Italy	8/293 (2.7)	-	-	-	-	-	-	-	-	*C. albicans* (3)<br>*C. glabrata* (3)<br>*C. parapsilosis* (2)<br>*C. krusei* (1)	-	4 (50)
Falcone [44]/ Italy	5/315 (1.6)	-	-	-	-	-	-	-	-	*C. parapsilosis* (5)	-	-
Fekkar [45]/ France	1/260 (0.4)	1 (100)/ 40	Obesity (1), AH (1)	-	10	1 (100)	-	-	1 (100)	*Candida* spp. (1)	Caspofungin (1)<br>Voriconazole (1)	0
Mastrangelo [128]/Italy	21/-	16 (76.2)/ 71 (57-75)	DM (2), CKD (4), IS therapy (11)	Steroids (8)	24 (12-28)	17 (80.9)	8 (38.1)	19 (90.5)	14 (66.7)	*C. albicans* (14)<br>C. non-*albicans* (7)	21 (100)	12 (57.1)
Cataldo [129]/ Italy	5/57 (8.8)	-	-	-	-	-	-	-	-	*C. albicans* (2)<br>*C. glabrata* (1)<br>*C. parapsilosis* (3)	-	-
Giacobbe [130]/ Italy	3/78 (3.8)	-	-	TCZ (2)	-	-	-	-	-	*C. albicans* (3)<br>*C. tropicalis* (1)	-	-

										C. parapsilosis (1)		
Nucci [131]/<br>Brazil	9/41<br>(21.9)	5 (55.6)/<br>62 (54-87)	DM (3), CVD (6), cancer (1), CKD (1), liver disease (1), neurological disease (2)	-	17 (0-22)	9 (100)	-	-	9 (100)	*C. albicans* (5)<br>*C. glabrata* (1)<br>*C. tropicalis* (2)<br>*C. famata* (1)	6 (66.7)<br>Anidulafungin (6)	6<br>(66.7)
Magnasco<br>[132]/<br>Italy	4/118<br>(3.4)	4 (100)/<br>63 (62-66)	Obesity (2),<br>CPD (1)	-	-	-	-	-	4 (100)	*C. auris* (4)	4 (100)<br>Caspofungin (4)<br>L-AMB (1)	2<br>(50)
Obata [51]/<br>USA	4/226<br>(1.8)	-	-	Steroids (4)<br>TCZ (3)	-	-	-	-	-	*Candida* spp. (4)	-	-
Ripa [2]/<br>Italy	7/731<br>(0.9)	-	-	-	-	-	-	-	-	*C. albicans* (5)<br>*C. parapsilosis* (2)	-	-
Versyck [54]/<br>France	3/54<br>(5.6)	-	-	-	-	-	-	-	-	*C. albicans* (2)<br>*C. tropicalis* (1)	-	-
Kayaaslan [15]/<br>Turkey	105/2487<br>(4.2)	55 (52.4)/<br>74 (60-81)	Obesity (52), DM (38), CVD (29), CPD (23), CKD (17), cancer (11), ID (6)	Steroids (67)	13 (8-24)	103 (98.1)	26 (24.8)	99 (94.3)	92 (87.6)	*Candida* spp. (11)<br>*C. albicans* (42)<br>*C. glabrata* (19)<br>*C. tropicalis* (13)<br>*C. parapsilosis* (20)	55 (52.4)<br>Anidulafungin (31)<br>Caspofungin (3)<br>L-AMB (1)<br>Micafungin (7)<br>Fluconazole (11)<br>Voriconazole (2)	97 (92.4)
Arastehfar<br>[133]/<br>Iran	7/1988<br>(0.4)	3 (42.9)/<br>68 (20-70)	DM (1), cancer (3)	-	6 (4-31)	7 (100)	7 (100)	7 (100)	5 (71.4)	*C. albicans* (5)<br>*C. glabrata* (3)<br>*R. mucilaginosa* (1)	7 (100)<br>Caspofungin (5)<br>Fluconazole (7)	6 (85.7)
Silva [134]/<br>Brazil	3/212<br>(1.4)	-	-	-	-	-	-	-	-	*C. albicans* (1)<br>*C. glabrata* (1)<br>*C. tropicalis* (1)	1 (33.3)<br>Fluconazole (1)	-
Signorini [60]/<br>Italy	41/92<br>(44.6)	-	-	-	-	-	-	-	-	*C. albicans* (33)<br>*Candida* non-*albicans* (8)	-	-
Seagle [14]/<br>USA	64/251<br>(25.5)	41 (64.1)/<br>-	Obesity (21), DM (34), CVD (21), CPD (12), HSCT (1), SOT (3), HCV (2), cancer (2)	Steroids (34)/<br>TCZ (57)	15 (8-21)	51 (79.7)	4 (6.3)	-	51 (79.7)	*C. albicans* (24)<br>*Candida* spp (1)<br>*C. glabrata* (17)<br>*C. tropicalis* (4)<br>*C. parapsilosis* (8)<br>*C. dubliniensis* (3)<br>*C. lusitaniae* (3)	53 (82.8)	40<br>(62.5)
Rodriguez<br>[135]/<br>Colombia	20/-	13 (65)/<br>63 mean	AH (11), DM (6), CKD (5), cancer (2)	Steroids (19)	17,7<br>(range 6-35)	19 (95)	2 (10)	20 (100)	19 (95)	*C. albicans* (2)<br>*C. glabrata* (1)<br>*C. tropicalis* (4)<br>*C. parapsilosis* (3)<br>*C. auris* (6)<br>*C.* orthopsilosis (1)<br>*T. asahii* (1)	15 (75)<br>Caspofungin (5)<br>Fluconazole (8)<br>Voriconazole (2)	12 (60)

Macauley [136]/ USA	12/236 (5.1)	9 (75)/ 62 +/- 16	DM (1), liver disease (1)	Steroids (6)	19 (11-28)	12 (100)	2 (16.7)	12 (100)	11 (91.7)	*C. albicans* (4)<br>*C. glabrata* (2)<br>*C. tropicalis* (2)<br>*C. parapsilosis* (3)<br>C. dubliniensis (1)<br>*Candida* non-*albicans* (1)	-	9 (75)

Data are number (percentage), unless otherwise specified.

Abbreviations. AH, arterial hypertension; DM, diabetes mellitus; CVD, cardiovascular disease; CPD, chronic pulmonary disease; SOT, solid organ transplant; ICU, intensive care unit; IQR, interquartile range; TI, tracheal intubation; CVC, central venous catheter; TPN, total parenteral nutrition; ATB, broad-spectrum antibiotics; IMV, invasive mechanical ventilation; CKD, chronic kindeny disease; AMB, amphotericin B; BARI, baricitinib; TCZ, tocilizumab; NS, not specified.
