# Supplementary material for: Invasive Fungal Infections Complicating COVID-19: A Narrative Review
Source: J Fungi (Basel). 2021 Oct 29;7(11):921. doi: 10.3390/jof7110921 (PMC8620819; doi:10.3390/jof7110921)
Supplement: Supplementary file 1 [file jof-07-00921-s001.zip › tables/Table S6 S7_Mucor reports and studies.pdf]

Table S6. Case report/case series describing mucormycosis associated with COVID-19.

Author/ Country	Cases	Sex/ Age	Underlyig diseases	Therapy COVID-19 (No. of patient)	During /Post COVID	Site	HP diagnosis	Positive culture, specimen	Species	Antifungal/ surgery	Outcome
Bellanger [84]/France	1	M/55	Cancer (1)	Steroids	During	Pulmonary	-	BAL	*R. microsporus*	AMB/No	Dead
Zurl [138]/ Austria	1	M/53	Obesity (1), AML with neutrope-nia(1)	Steroids TCZ	During	Pulmonary, pleural effusion	Autopsy	No	*R. microsporus*	No/No	Dead
Garg [139]/ India	1	M/55	DM (1), CVD (1), CKD (1)	Steroids	During	Pulmonary	Sputum	Sputum	*R. microsporus*	AMB/No	Alive
Johnson [88]/ USA	1	M/79	AH (1), DM (1)	-	During	Pulmonary	BAL	BAL	*R. arrhizus*	AMB/No	Alive
Khatri [140]/ USA	1	M/68	AH (1), DM (1), CVD (1), SOT (1), steroids (1), CKD (1)	-	Post	Soft tissues, blood vessels wall	Pleural effusion	Pleural effusion Soft tissues	*R. microsporus*	Posaconazole AMB/ Yes	Dead
Pasero [141]/ Italy	1	M/66	AH (1)	-	During	Pulmonary	BAL	BAL	*Rhizopus* spp.	Isavuconazole AMB/No	Dead
Arana [142]/ Spain	2	M/62	AH,DM, CKD, CVD, SOT	Steroids	During	Rhino-sinusal	Nasal biopsy	Nasal swab	*R. arrhizus*	Isavuconazole AMB/Yes	Alive
		M/48	AH, CKD, SOT	Steroids	During	Muscolo-skeletal	Muscle biopsy	Muscle biopsy	*L. ramosa*	Isavuconazole AMB/ Yes	Alive
Rao [143]/ India	1	M/66	DM	Steroids	During	ROC	Nasal swab	No	-	AMB/Yes	Alive
Krishna [144]/ UK	1	M/22	Obesity	Steroids	During	Disseminated	Autopsy	No	*Mucorales* spp. (PCR)	No/No	Dead
Meshram [145]/ India	2	M/45	AH, DM, SOT, chronic steroid	No	Post	RO	Nasal biopsy	No	-	AMB/Yes	Dead
		M/23	AH, DM, SOT, chronic steroids	No	Post	Pulmonary	BAL + lung biopsy	No	-	AMB/Yes	Dead
Nehara [146]/ India	5	F/59	AH, DM	-	During	ROC	Nasal discharge	Nasal discharge	*Rhizopus* spp.	AMB/No	Dead
		M/52	DM	-	During	ROC	Nasal discharge	Nasal discharge	*R. arrhizus*	AMB Posaconazole/ No	Alive
		F/62	AH, DM	-	During	ROC	Nasal scraping	Nasal scraping	*Rhizopus* spp.	AMB/Yes	Alive

		F/70	DM	-	During	ROC	Nasal scraping	Nasal scraping	*R. arrhizus*	AMB/No	Alive
		F/68	DM	-	During	ROC	Nasal scraping	Nasal scraping	*R. arrhizus*	AMB/No	Dead
Buil [147]/ Netherland	4	M/60s	No	Steroid TCZ	During	Pulmonary	-	Sputum	*R. microsporus*	Voriconazole AMB Posaconazole/No	Alive
	+CAPA	M/50s	No	Steroid TCZ	During	Pulmonary	-	BAL	*L. ramosa*	Voriconazole AMB Posaconazole/No	Dead
	+CAPA	M/60s	CLL, DM, obesity	Steroid TCZ	During	Pulmonary	Autopsy	BAL	*R. microsporus*	Voriconazole Anidulafungin Isavuconazole Voriconazole/No AMB	Dead
		M/70s	DM, chronic steroid	Steroid	Post	ROC	-	Orbital purulent material	*R. arrhizus*	AMB Isavuconazole/Yes	Dead
Ashour [148]/ Egypt	7	F/65	DM	-	During	ROC	Nasal biopsy	Nasal biopsy	NS	AMB Itraconazole/Yes	Alive
		M/67	DM, CKD	-	During	ROC	Nasal biopsy	No	-	AMB/No	Dead
		M/42	DM, CVD	-	During	Rhino-sinusal	Nasal biopsy	No	-	AMB/Yes	Alive
		F/63	DM	-	Post	ROC	Nasal biopsy	Nasal biopsy	-	Yes/Yes	Alive
		F/41	DM	-	During	ROC	Nasal biopsy	No	-	AMB/Yes	Alive
		M/54	AH, DM, CKD, CVD	-	During	Rhino-sinusal	Nasal biopsy	No	-	Yes/No	Dead
		M/50	DM	-	Post	ROC	Nasal biopsy	No	-	Yes/Yes	Dead
Kanwar [149]/USA	1	M/56	CKD	Steroids TCZ	Post	Pulmonary	Pleural biopsy	Pleural effusion	*R. azygosporus*	AMB/Yes	Dead
Placik [150]/ USA	1	M/49	No	Steroids TCZ	During	Pulmonary (broncopleural fistula)	Lung biopsy	Pulmonary tissue	*Rhizopus* spp.	AMB/Yes	Dead
Veisi[151]/ Iran	2	F/40	No	Steroids	During	ROC	Nasal biopsy	No	-	AMB (iv and topical)/Yes	Dead
		M/54	DM	Steroids	During	RO	Nasal biopsy	No	-	AMB Posaconazole/Yes	Alive
Saldanha [152]/India	1	F/42	DM	-	During	ROC	Nasal biopsy	No	-	AMB/Yes	Alive
Revannavar [153]/India	1	F/-	DM	-	During	ROC	Nasal biopsy	Nasal biopsy	*Rhizopus* spp.	AMB/Yes	Alive
Maini [154]/ India	1	M/38	No	Steroids	During	RO	Nasal biopsy	Nasal biopsy	*R. arrhizus*	AMB Fluconazole/Yes	Alive
Alekseyev [155]/USA	1	M/41	DM	Steroids	During	ROC	Nasal biopsy	No	-	AMB/Yes	Alive
Krishna [156]/India	2	M/34	AH, DM	-	During	RO	Nasal biopsy	No	-	AMB Itraconazole/Yes	Alive
		M/50	DM	-	During	RO	Nasal biopsy	No	-	AMB Posaconazole/Yes	Alive

Werthman-Ehreeich [157]/USA	1	F/33	AH, DM, CPD	-	During	RO	Nasal biopsy	Nasal biopsy	NS	AMB/Yes	Dead
Hanley [158]/ UK	1	M/22	Obesity, panchreatitis	-	During	Disseminated	Autopsy	No	-	No/No	Dead
Mehta [159]/ India	1	M/60	DM, CVD	Steroids TCZ	During	RO	Nasal biopsy	Nasal biopsy	NS	AMB/Yes	Dead
do Monte-Junior [160]/ Brazil	1	M/86	AH	Steroids	During	Gastric	Gastric biopsy	No	-	No/No	Dead
Mekonnen [161]/ USA	1	M/60	AH, DM, CPD	Steroids	During	RO	Nasal biopsy	Nasal biopsy	*Rhizopus* spp.	AMB (iv and topical) Posaconazole Caspofungin/Yes	Dead
Selarka [162]/ India	1	M/42	DM	Steroids	Post	RO	Nasal aspirate	No	-	AMB/Yes	Alive
Sarkar [163]/ India	10	M/67	DM	Steroids	During	RO	Nasal biopsy	Negative	No	AMB/Yes	Alive
		M/49	DM	Steroids	During	RO	Nasal biopsy	Negative	No	AMB/No	Dead
		M/23	DM	Steroids	During	RO	Nasal biopsy	Nasal biopsy	*Rhizopus* spp.	AMB/Yes	Dead
		F/59	DM	Steroids	During	RO	Nasal swab	Nasal swab	*Mucor* spp.	AMB/No	Dead
		F/27	DM	Steroids	During	RO	Nasal biopsy	Nasal biopsy	*Rhizopus* spp.	AMB/Yes	Alive
		M/45	DM	Steroids	During	RO	Nasal biopsy	Nasal biopsy	*Mucor* spp.	AMB/Yes	Alive
		M/48	DM	Steroids	During	ROC	Nasal biopsy	Nasal swab	*Rhizopus* spp.	AMB/No	Dead
		M/62	DM	Steroids	During	RO	Nasal biopsy	Nasal biopsy	*Rhizopus* spp.	AMB/yes	Alive
		M/43	DM	Steroids	During	RO	Nasal biopsy	Negative	No	AMB/yes	Alive
		M/32	DM	Steroids	During	RO	Nasal biopsy	Negative	No	AMB/yes	Alive
Karimi-Galougahi [164]/India	1	F/61	DM	Steroids	Post	RO	Nasal biopsy	No	-	AMB/Yes	Alive
Dallalzadeh [165]/USA	1	M/48	DM	Steroids	During	ROC	No	Purulent orbital fluid	*Rhizopus* spp.	AMB Isavuconazole/No	Dead
Weizel-Haiat [166]/Mexico	1	F/24	Obesity, DM	-	During	RO	Nasal swab	Nasal swab	*Lichthemia* spp.	AMB/No	Dead
Khan]167]/ USA	1	F/44	DM	Steroids	During	Pulmonary	BAL	No	-	AMB Voriconazole Micafungin/No	Dead
Pauli [168]/ Brazil	1	F/50	DM	-	During	Palatal	Nasal biopsy	No	-	AMB/Yes	No

Data are number (percentage), unless otherwise specified.

Abbreviations. AH, arterial hypertension; DM, diabetes mellitus; CVD, cardiovascular disease; CPD, chronic pulmonary disease; SOT, solid organ transplant; ICU, intensive care unit; IQR, interquartile range; TI, tracheal intubation; CKD, chronic kindeny disease; AML, acute myeloid leukemia; CLL, chronic lymphocytic leukemia; ID, immunedepression; SOT; solid organ transplantation; AMB, amphotericin B; TCZ, tocilizumab; RO, rhino-orbital; ROC; rhino-orbital-cerebral; NS, not specified; NR, not reported; BAL, bronchial alveolar alavage; HP, histopathological.

Table S7. Observational studies describing mucormycosis associated with COVID-19.

Author/ Country	No. of cases/ total (%)	Males (%)/ Median age yrs (IQR)	Underlying diseases	Therapy COVID-19 (No. of patient)	During/ Post COVID	Site (%)	No. of HP diagnosis (%)	Positive culture, No. (%)	Species (No.)	Patients treated (%)/Anti-fungal agents used (No.)	Surgery/ No. of patients (%)	Deaths (%)
Fekkar [45]/ France	2/260 (0.8)	2 (100)/ 50 & 75 years	Obesity (2), AH (1), SOT (1)	-	2/0	Pulmonary (2)	0	0/2	*Rhizopus*/ *Mucor* (2; positive PCR on RS)	1 (50) VRC (1) ISA (1) AMB (1) CAS (1)	0	1 (50)
Ravani [169]/ India	19/31 (61.3)	-	-	Steroids (19)	0/19	RO (19), of which 7 with cerebral involvement	19 (100)	0	NS (19)	19 (100) POS (19) AMB (19)	19 (100)	-
Sen [170]/ India	6/-	6 (100)/ 61 (47-72)	AH (83), DM (6), CVD (2)	Steroids (5)	1/5	RO (1) ROC (5)	4 (80)	3 (50)	*Mucor* spp. (6)	6 (100) POS (5) VRC (1) AMB (2), 1 topic	6 (100)	0
Pakdel [171]/ Iran	15/58 (25.9)	10 (66.7)/ 52 (14-71)	AH (7), DM (13), CVD (2), CPD (2), cancer (2), cirrhosis (1), neutropenia (3)	Steroids (7)	15/0	RO (12) Orbital (2) Rhino-sinusal (1)	15 (100)	15 (100)	NS (15)	15 (100) POS (4) AMB (15) CAS (3)	14 (93.3)	7 (46.7)
Bayram [18]/ India	11/ 32'814 (0.03)	9 (81.8)/ 73 (68-79)	AH (5), DM (2), CVD (3), CKD (2), MDS (1)	Steroids (11) TCZ (2)	11/0	RO (8) ROC (3)	11 (100)	11 (100)	*Rhizopus* spp. (11)	11 (100) AMB iv + topic (11)	11 (100)	7 (63.6)
Moorthy [172]/ India	17/18 (94.4)	15 (83.3)/ 56 (47-63)	DM (15)	Steroids (15)	4/13	RO (6) ROC (5) Rhino-sinusal (3) Rhino-cerebral (3)	17 (100) + 1 *Aspergillus*	-	NS (17)	17 (100) AMB (17)	16 (94.1)	6 (35.5)
Sharma [173]/ India	23/-	15 (65.2)/ NR	AH (14), DM (21), CKD (1)	Steroids (23)	4/19	RO (8) ROC (2) Rhino-sinusal (13)	23 (100)	-	NS (23)	23 (100) AMB (23; topic application in 2 cases)	23 (100)	0
Ismaiel [174]/ Egypt	12/29 (41.4)	-	DM (8), CKD (1), IS 81)	-	0/12	RO (12)	12 (100)	12 (100)	*R. arrhizus* (8) *Lichtemia* (2) NS (2)	-	-	-
Fouad [175]/ Egypt	6/12 (50)	4 (66.7)/ 54 (42-63)	DM (6), CVD (1), CKD (2)	Steroids (3)	5/1	RO (3) ROC (3)	6 (100)	0	-	6 (100)	3 (50)	3 (50)

Patel [176]/ India	187/ 287 (65.2)	150 (80.2)/ 57 +/- 12	DM (113), cancer (2), SOT (3)	Steroids (150) TCZ (5)	Early 29, late 158*	Pulmonar (16) RO (117) ROC (44) Disseminated (4) Renal (1) NS (5)	143 (76.5)	100 (53.5)	*Mucorales* (99) *Mucorales* + *Aspergillus* (1)	187 (100) POS (73) ISA (19) AMB (136)	131 (70.1)	75 (40.1)
Sen [177]/ India	2826/-	1993 (71)/ 53 (12-88)	DM (2194), AH (690), CKD (88), obesity (2), CPD (17), CVD (24), HIV (4), SOT (4)	Steroids (2073) TCZ (58)	Median 13 days since COVID diagnosis	Rhino-sinusal (752) RO (1344) ROC (573)	1090/2795 (39)	432/2175(19) 1931/2175 with positive DE	NS (432)	2066 (73) AMB (2066) POS (698) ISA (34)	1585/2358 FESS (67), of which 367 did orbital exenteration	305/2218(14)
Rabagliati [61]/ Chile	2/146 (1.4)	2 (100)/ 58.5 median	AH (1), DM (1), CVD (1)	Steroids (1)	2/0	Pulmonary (2), of which 1 with cerebral involvement	0	2 (100)	*R. stolonifer* (1) *R. microsporus* (1)	1 (50) AMB (1)	0	1 (50)

Data are number (percentage), unless otherwise specified.

Abbreviations. AH, arterial hypertension; DM, diabetes mellitus; CVD, cardiovascular disease; CPD, chronic pulmonary disease; SOT, solid organ transplant; ICU, intensive care unit; IQR, interquartile range; TI, tracheal intubation; CKD, chronic kindeny disease; SOT; solid organ transplantation; MDS, myelodisplastic syndrome; AMB, amphotericin B; TCZ, tocilizumab; RO, rhino-orbital; ROC; rhino-orbital-cerebral; NS, not specified; NR, not reported; BAL, bronchial alveolar lavage; CT, chemotherapy; TA, tracheal aspirate; HP, histopathological; VRC, voriconazole; ISA, isavuconazole; CAS, caspofungin; POS, Posaconazole; RS, respiratory sample; DE, direct examination of respiratory samples.
* cut-off time: 8 days since COVID-19 diagnosis.
